# Effect of Impact Parameters on a Unilateral Contusion Model of Spinal Cord Injury in a Virtual Population of Non-Human Primates

**DOI:** 10.1089/neur.2023.0006

**Published:** 2023-06-01

**Authors:** Numaira Obaid, Ana-Maria Bojic, Shervin Jannesar, Ernesto Salegio, Yvette Nout-Lomas, Michael Beattie, Jacqueline Bresnahan, Carolyn Sparrey

**Affiliations:** ^1^ Mechatronic Systems Engineering, Simon Fraser University, Surrey, British Columbia, Canada.; ^2^ International Collaboration on Repair Discoveries (ICORD), Vancouver, British Columbia, Canada.; ^3^ Biomedical Physiology and Kinesiology, Simon Fraser University, Burnaby, British Columbia, Canada.; ^4^ School of Engineering Science, Simon Fraser University, Burnaby, British Columbia, Canada.; ^5^ University of California, Davis, California, USA.; ^6^ Department of Clinical Sciences, Colorado State University, Fort Collins, Colorado, USA.; ^7^ Brain and Spinal Injury Center, Department of Neurological Surgery, University of California, San Francisco, California, USA.

**Keywords:** animal models, computational models, contusion, spinal cord injury, variability

## Abstract

Non-human primate (NHP) spinal cord injury experiments exhibit high intersubject variability in biomechanical parameters even when a consistent impact protocol is applied to each subject. Optimizing impact parameters to reduce this variability through experiments is logistically challenging in NHP studies. Finite element models provide a complimentary tool to inform experimental design without the cost and complexity of live animal studies. A morphologically variable virtual population (*N* = 10) of NHPs quantified the interaction of morphological variability and different impact conditions in a unilateral cervical contusion, including impactor size (4 and 5 mm) and mediolateral alignment over the cord midline (0.5 and 1 mm). We explored the effect of these conditions on the magnitude and intersubject variability of impact force and cord lateral slippage. The study demonstrated that a 1-mm mediolateral alignment maximized peak forces and minimized lateral slippage. A 5-mm impactor was beneficial in increasing peak forces, whereas a 4-mm impactor reduced lateral slippage. Comparatively, intersubject variability in peak forces and lateral slippage were minimized with a 0.5-mm mediolateral alignment. The study highlights that impact parameters selected based on peak forces may not be beneficial in reducing variability. The study also showed that morphology was an important contributor to variability. Integrating morphology variability through a virtual population in an injury simulation to investigate mechanistic research questions will more effectively capture the heterogeneity of experiments and provide better insights for effective experimental design.

## Introduction

Experimental studies exploring spinal cord injuries (SCIs) in large animal models show that morphological differences between study subjects led to variability in tissue damage and functional outcomes observed after injury.^1–3^ It is important to ensure that this experimental variability represents the inherent heterogeneity of SCIs and not a lack of reproducibility because of the selection of impact parameters. The impact protocols used in animal models need to be selected to ensure that the delivered contusion produces an injury that is sufficiently severe to observe treatment effects but moderate enough to ensure that the animal can be effectively cared for over the duration of the study. Limiting the injury to the contralateral cord is particularly important for long-term studies where ongoing care is required post-injury.

Examining the effect of impact parameters in experimental studies is difficult in large animals, particularly in non-human primates (NHPs), because it would require a high number of subjects, which is ethically challenging and cost-prohibitive. In the past, impact protocols for large animal models have been developed by scaling parameters from rodent studies and predicting outcomes using computational models of SCI.^3^ Computational models, which can predict tissue-level loading (stress) and deformation (strain) experienced by the cord while a contusion injury is delivered, have been used in past studies.^4–6^ For example, Khuyagbaatar and colleagues examined the effect of impactor size on the biomechanics of a rat cervical hemicontusion model.^7^ The study identified that there was a preferred impactor size among the selected options, where using an impactor that is too small results in low peak forces whereas using an overly large impactor causes peak forces to spread to the contralateral side. The study deemed impactor size to be an important parameter in animal SCI models.

Large animals, such as NHPs, display significant morphological variability in the spinal cord and column, and this morphological variability affects SCI outcomes.^1^ However, despite its significance, morphological variability is not incorporated into most computational studies, where they use a single generic model to draw conclusions about a variable population. Capturing the morphological variability observed in real animal subjects is important for representing the heterogeneous nature of SCI observed in animal and human experiments. Incorporating morphological variability into computational models will increase the robustness and relevance of these simulations by mirroring the variability observed in experiments.

The purpose of the study was to examine the effect of impact parameters in a unilateral contusion model of an NHP SCI computationally, while capturing the population heterogeneity observed in real experiments. In this study, we present a novel approach, where the morphological variability of the NHP will be incorporated into a computational SCI model. A virtual population of NHPs was used to examine the effect of impactor size and its mediolateral alignment over the cord midline on the biomechanical outcomes and variability in a unilateral cervical contusion injury.

## Methods

**FIG. 1.**
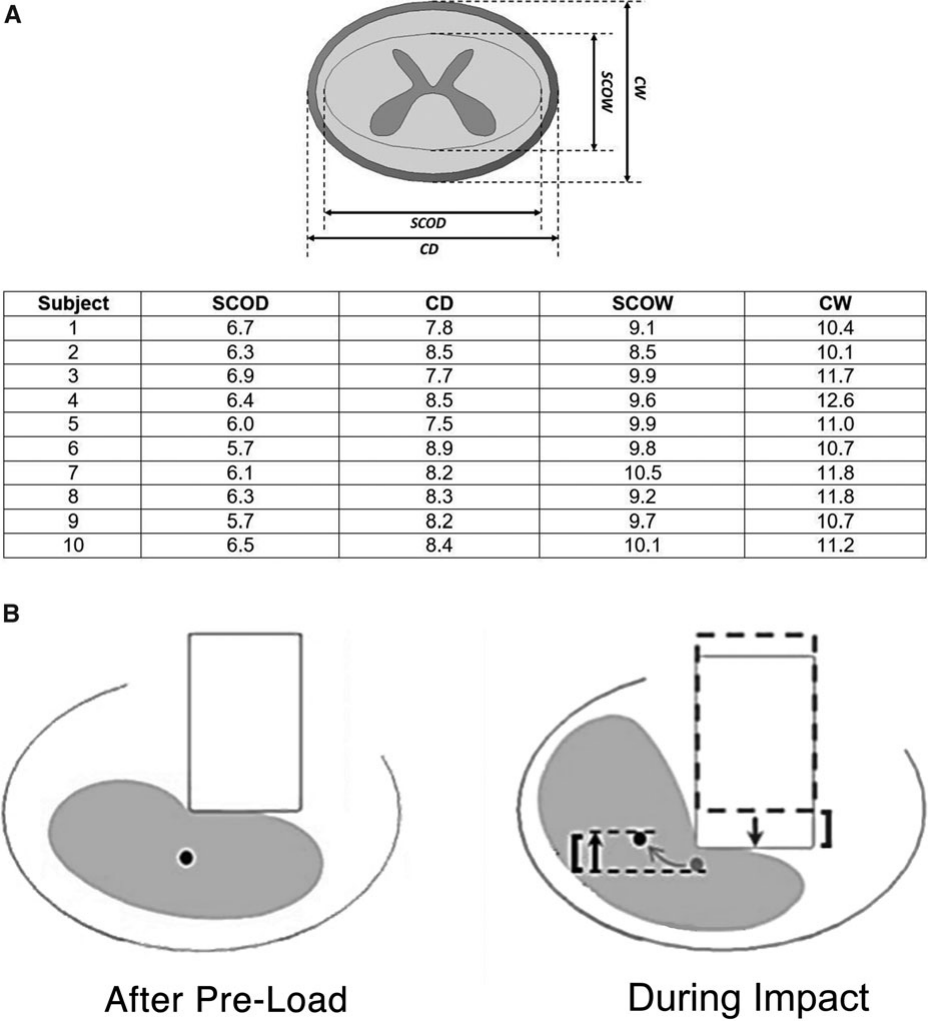
(**A**) Virtual subjects used in this study varied in their spinal cord depth and width (SCOD and SCOW) and spinal canal depth and width (SCD and SCW) in millimeters. These parameters are visually represented here. Cervical spine and spinal cord morphology of the virtual subjects used in this study are listed. (**B**) Lateral slippage was quantified as the relative vertical position of the cord midpoint compared to the impactor at peak impact. Rotation and lateral slippage of the cord causes the cord midpoint to be located higher than one with lower lateral slippage. A higher relative position was assumed to represent increased lateral slippage.

A python code was used to generate 10 virtual NHP geometries using the Abaqus Scripting Interface (ASI). The code randomly selected values for each subject's anterior-posterior cord and canal diameter (spinal cord depth [SCOD] and spinal canal depth [SCD], respectively) and mediolateral cord and canal diameter (spinal cord width [SCOW] and spinal canal width [SCW], respectively; see Fig. 1A). The upper and lower bound of values for each parameter inputted into the python code were based on 14 *Macaca mulatta* cervical spine T2 magnetic resonance images (MRIs; 3T Seimens MAGNETOM Skyra with custom four-channel Clamshell MRI coil; Siemens Healthcare, Erlangen, Germany).^6,8^

A unilateral contusion was simulated using finite element analysis (Abaqus 2021; Simulia Inc., Johnston, RI). The model included a homogeneous spinal cord (gray and white matters and pia mater), cerebrospinal fluid (CSF), dura mater, and the spinal canal of a cervical spine from C4 to C6. The canal and impactor were defined as rigid bodies, and the dura was defined as a shell (*t* = 350 μm).^5^ The C5 was modified to represent a partial laminectomy of 12 mm by 12 mm.^8^ A cylindrical impactor delivered a contusion at the C5 level, positioned relative to the spinal cord midline. The CSF was represented using smoothed particle hydrodynamics.^5^ The properties of the dura were extracted from our earlier studies,^4,9^ CSF properties were from Jannesar and colleagues,^5^ and spinal cord properties were obtained from the literature.^10^

The contusion impact was simulated to mimic our NHP unilateral cervical contusion experiments^4–6^ by two dynamic, explicit steps. First, a pre-load phase, where the CSF was slowly displaced by the impactor until the cord was entrapped against the spinal canal until the pre-load force reached 0.4 N for a 4-mm impactor or 0.75 N for a 5-mm impactor. A pre-load is used in experimental NHP models to displace the CSF and limit the contralateral involvement of the spinal cord during the contusion injury.^8^ Second, an impact phase, where a high-speed contusion (∼500 mm/s to a 4-mm displacement) was delivered.^5,8^ During the pre-load phase, only the rostral-caudal movement of cord and dura was restricted. The ends of the cord and dura were fully constrained during the impact phase. The CSF was allowed to flow in the rostral-caudal direction during the pre-load phase, but was blocked during the impact phase, representing the pressurization of the CSF during a high-speed impact. These boundary conditions were validated in previous computational models of SCI.^4,5,9^ Contacts were defined based on validated penalty coefficients.^5^

Peak impact forces and cord lateral slippage were quantified as the primary outcomes. Impact forces were compared against experimental results to confirm the validity of this modeling approach. In this study, we calculated lateral slippage as the relative vertical position of the cord midpoint directly under the impactor compared to the vertical position of the impactor at peak impact time (see Fig. 1B). This assumption was validated by comparing measured values to the deformed shape of the cord examined visually, that is, if the cord with the maximum cord slippage upon visual examination had the highest relative position.

We examined the effect of changing impactor size and mediolateral alignment on peak force and lateral movement using four scenarios: 1) 4-mm diameter impactor aligned 0.5 mm over midline; 2) 5-mm impactor aligned 0.5 mm over midline; 3) 4-mm impactor aligned 1 mm over midline; and 4) 5-mm impactor aligned 1 mm over midline. These values were selected based on the current experimental protocols used to induce NHP contusions.^3,8^

## Statistical analysis

Pearson correlation coefficients were also computed to determine which morphological parameters contributed to the biomechanical outcomes and variability. Any correlation coefficient with a magnitude >0.6 was considered important. The study investigated correlations with the main morphological parameters (SCOD, CD, SCOW, and CW), but also their inter-relationships including the occlusion of the canal by the cord in the anterior-posterior direction (SCOD/CD) and the mediolateral direction (SCOW/CW), as well as the cross-sectional area of the CSF (canal cross-sectional area minus spinal cord cross-sectional area) in the transverse direction.

## Results

Peak forces increased with increasing impactor diameter or mediolateral alignment, with an average increase of 15% for each variable, and increasing both simultaneously increased peak forces by 36%. The highest average peak impact force was obtained using an impactor with a 5-mm diameter, aligned 1 mm over the cord midline (21.3 N), whereas a 4-mm impactor diameter aligned 0.5 mm over the cord midline resulted in the lowest average peak impact force (15.7 N). Though the difference is small, at both mediolateral alignments, a 5-mm impactor resulted in lower intersubject variability (9.7% for a 0.5-mm alignment and 11.8% for a 1-mm alignment) in peak forces compared to a 4-mm impactor (10.7% for a 0.5-mm alignment and 12.1% for a 1-mm alignment). Regardless of impactor diameter, increasing the mediolateral alignment of the impactor from 0.5 to 1 mm increased intersubject variability in the peak force.

**FIG. 2.**
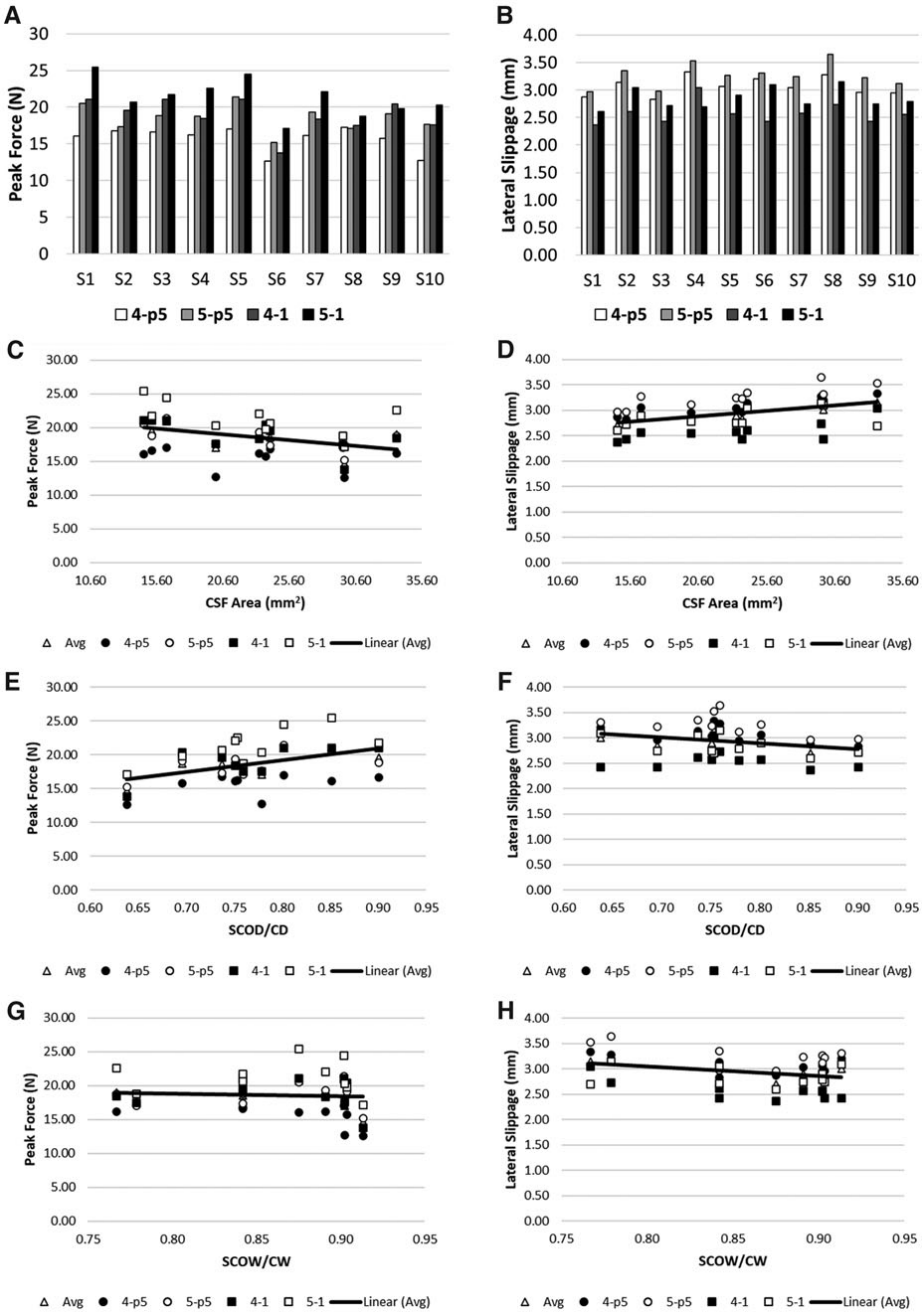
Peak force (**A**) and lateral slippage (**B**) of the cord varied under different impact conditions and were calculated for each subject. Results are presented here (**C–H**) for three different morphological parameters: the CSF area and the occupancy ratio of the canal area by the cord in the anterior-posterior and mediolateral axes (SCOD/SCD and SCOW/SCW, respectively). CSF, cerebrospinal fluid.

Peak impact forces varied for each subject (Fig. 2A**)** and ranged from 12.6 to 25.4 N in the simulations compared with forces of 12.5–23.2 N in the experiments. Subject 6 consistently had the lowest impact forces at all impact parameters. This could be attributed to this subject having the least occlusion of the spinal cord in the spinal canal (lowest SCOD/SCD) ratio, which could have increased slippage during the pre-load phase. For most subjects, a 5-mm diameter impactor aligned 1 mm over the midline produced the highest peak forces. The only exception was subject 9, where the highest peak forces were produced by a 4-mm impactor aligned 1 mm over the cord midline; however, this was only 3% higher than a 5-mm impactor with the same alignment.

Subjects also demonstrated different sensitivity toward changes in impact parameters. For example, subject 1 was the most sensitive to changes in the impact parameters (45% change) whereas subject 8 was the least affected by changes in impact parameters (9.5% change). The increased sensitivity of subject 1 to impact parameters could be attributed to having had the lowest CSF area whereas subject 8, which was least affected by impact parameter changes, had the highest CSF area.

Lateral slippage of the cord during impact was also quantified for each subject (Fig. 2B). A 4-mm diameter impactor aligned 1 mm over the midline minimized the magnitude of lateral slippage. For all subjects, increasing mediolateral alignment was beneficial in reducing lateral slippage. Similarly, increasing impactor size led to increased lateral slippage. This is possibly because the 4-mm impactor entrapped larger sections of the dura on both the right and left side of the impactor, whereas a 5-mm impactor had a lower ratio of the dura entrapped on the lateral side of the impactor.

Peak forces observed during the impact were sensitive to occlusion of the spinal canal by the cord in the dorsoventral direction (SCOD/SCD; *r* = 0.67) as well as the area of the CSF (*r* = −0.63). A higher CSF area reduced peak forces, whereas increased occupancy of the canal by the cord resulted in higher peak forces. Increasing the area of the CSF also increased lateral slippage of the cord (*r* = 0.81). Lateral slippage of the cord was also affected by mediolateral occupancy of the canal by the cord (SCOW/SCW; *r* = −0.69), where reducing the occupancy increased lateral slippage.

## Discussion

Morphological differences produce variability in biomechanical and functional outcomes in contusion models of large animal SCI.^1,5^ These differences are not currently represented in computational models of SCI, which limits the translation of findings if the models do not adequately represent the heterogeneity of injury observed in real experiments. In this study, we present a novel method to incorporate morphological differences into computational models of SCI and evaluate the effect of changing impact parameters using a virtual population.

The statistical analysis showed that increasing the CSF area reduced peak forces. This was expected because the CSF layer has a protective effect on the cord^11^ and increasing its thickness would allow it to dissipate more energy from the impact, generating lower forces. This also agrees with findings in experimental studies conducted in large animals, where the CSF has been identified as an important morphological variable contributing to injury heterogeneity.^1^ A thicker CSF also increased cord slippage. This is also expected because a thicker CSF layer would mean that the cord occupies less space within the canal, providing it with additional space for lateral movement and rotation. Conversely, a spinal cord that occupies a larger fraction of the canal has less space available for lateral movement.

Applying different impact protocols also had different effects on each subject in the study, with some subjects being more sensitive to changes in the impactor diameter and mediolateral alignment whereas others were more resilient to these changes. This sensitivity increased with decreased CSF in the subject. Subject 1, with the lowest amount of CSF, was the most sensitive to changes in peak force magnitude with changes in the impactor parameters, whereas subject 8, with the highest amount of CSF, was least sensitive to the changes. This is perhaps because a higher CSF area allows for more lateral motion of the spinal cord during impact, whereas a reduced CSF area mechanically constrains the cord from lateral motion. This was also evident by subject 1 having the least lateral movement of the spinal cord. Therefore, subjects with lower CSF not only experienced higher peak forces, but were also more susceptible to changes in peak forces with changes in the impact parameters.

Increasing mediolateral alignment from 0.5 to 1 mm and increasing impactor size from 4 to 5 mm had similar effects of increasing average peak force. For 9 of 10 subjects, using a 5-mm impactor aligned 1 mm over the cord midline generated the highest peak forces. This finding agrees with previous studies demonstrating that increasing mediolateral alignment and impactor diameter increases peak forces.^3^ To create more severe injuries, a 5-mm impactor is advantageous over a 4-mm impactor, and a mediolateral alignment of 1 mm over midline is better than a mediolateral alignment of 0.5 mm.

The impact protocols also altered variability in peak forces within the subset, with the highest intersubject variability in peak forces being produced by a 4-mm impactor aligned 1.0 mm over the cord midline and lowest for a 5-mm impactor with a 0.5-mm alignment. Regardless of impactor size, a mediolateral alignment of 0.5 mm was more advantageous in reducing peak force variability than a 1-mm alignment. This is possibly because a 0.5-mm impactor would pinch the lateral end of the cord and entrap it against the canal, preventing further lateral slippage. This is further substantiated by the 0.5-mm mediolateral alignment reducing intersubject variability in lateral slippage compared to a 1-mm alignment.

The selected impact protocol used to deliver contusions should optimize the creation of injuries that can be used to detect treatment effects, but also consider the potential variability in expected outcomes from a set of animal subjects. For example, whereas the lowest lateral slippage and variability occurs for a 4-mm impactor aligned 1 mm over the cord midline, a 4-mm impactor generates ∼11% lower average peak forces than a 5-mm impactor. In some subjects, this difference can be higher; for example, in subject 6, switching from a 5- to a 4-mm impactor aligned 1 mm over the midline reduced peak forces by ∼20%. This variability between subjects can change outcomes significantly if a 20% higher peak force leads to more severe injuries in this subject. The main factor influencing the range of peak forces across the observed impactor conditions was the area of the CSF in the subject, where subjects with a lower CSF area displayed a wider range of peak forces with changing impact conditions (*r* = −0.6) and reduced lateral slippage (*r* = 0.85).

## Conclusion

We present the first attempt to incorporate intersubject variability into the simulations of a unilateral contusion model of SCI using a virtual population of NHPs. The study quantified the effect of morphology on biomechanical outcomes, and found that the area of the CSF is an important contributor to outcome variability. Peak forces were maximized using a 5-mm impactor aligned 1 mm over the cord midline whereas the amount of lateral slippage was minimized using a 4-mm impactor aligned 1 mm over the midline. Intersubject variability in peak forces was minimized by using a 5-mm impactor aligned 0.5 mm over the cord midline, and the lowest variability in lateral slippage was observed for a 4-mm impactor aligned 0.5 mm over the cord midline.

This study quantified how different impact parameters affected the magnitude of biomechanical outcomes and their variability while incorporating intersubject morphological variability into the model. We showed that subjects with a lower area of CSF were less sensitive to lateral slippage, but their peak forces were more sensitive to changes in the impactor parameters. Conversely, a higher CSF area resulted in increased lateral slippage, but less sensitivity to impactor parameters. The study further emphasized the important role of a subject's morphology on the phenomena that occur during a unilateral contusion impact.

This approach to injury simulation has the potential to be applied to investigate mechanistic research questions incorporating large numbers of subjects computationally to more effectively capture the heterogeneity of experiments and provide better insights for effective experimental design. For example, in this study, if the computational model had only investigated the effect of impactor parameters on peak forces in subject 9, we would have concluded that the peak forces were maximized using a 4-mm impactor with a 1-mm mediolateral alignment, whereas using any other subject would have concluded that peak forces were maximized with a 5-mm impactor with a 1-mm mediolateral alignment. Incorporating intersubject variability increases the validity of computational models of SCI in accurately capturing the range of experimental outcomes.

## Abbreviations Used

CSF: cerebrospinal fluid
MRI: magnetic resonance imaging
NHP: non-human primate
SCD: spinal canal depth
SCI: spinal cord injury
SCOD: spinal cord depth
SCOW: spinal cord width
SCW: spinal canal width
